# Ad5/3 is able to avoid neutralization by binding to erythrocytes and lymphocytes

**DOI:** 10.1038/s41417-020-00226-z

**Published:** 2020-09-12

**Authors:** Sadia Zafar, Dafne Carolina Alves Quixabeira, Tatiana Viktorovna Kudling, Victor Cervera-Carrascon, Joao Manuel Santos, Susanna Grönberg-Vähä-Koskela, Fang Zhao, Pasi Aronen, Camilla Heiniö, Riikka Havunen, Suvi Sorsa, Anna Kanerva, Akseli Hemminki

**Affiliations:** 1 Cancer Gene Therapy Group, Translational Immunology Research Program and Department of Oncology, University of Helsinki, Helsinki, Finland; 2 TILT Biotherapeutics Ltd, Helsinki, Finland; 3 Advanced Microscopy Unit (AMU), Faculty of Medicine, University of Helsinki, Helsinki, Finland; 4 Biostatistics Unit, Faculty of Medicine, University of Helsinki, Helsinki, Finland; 5 Department of Obstetrics and Gynecology, Helsinki University Hospital, Helsinki, Finland; 6 Helsinki University Hospital Comprehensive Cancer Center, Helsinki, Finland

**Keywords:** Cancer, Immunology

## Abstract

Oncolytic adenoviruses are promising cancer therapeutic agents. Clinical data have shown adenoviruses’ ability to transduce tumors after systemic delivery in human cancer patients, despite antibodies. In the present work, we have focused on the interaction of a chimeric adenovirus Ad5/3 with human lymphocytes and human erythrocytes. Ad5/3 binding with human lymphocytes and erythrocytes was observed to occur in a reversible manner, which allowed viral transduction of tumors, and oncolytic potency of Ad5/3 in vitro and in vivo*,* with or without neutralizing antibodies. Immunodeficient mice bearing xenograft tumors showed enhanced tumor transduction following systemic administration, when Ad5/3 virus was bound to lymphocytes or erythrocytes (*P* < 0.05). In conclusion, our findings reveal that chimeric Ad5/3 adenovirus reaches non-injected tumors in the presence of neutralizing antibodies: it occurs through reversible binding to lymphocytes and erythrocytes.

## Introduction

Adenoviruses are well characterized and efficient gene-delivery vectors. They offer broad potential for therapy and can be used as oncolytic agents. There are more than 50 serotypes of adenovirus have been described [1]. For gene therapy applications, the most commonly used is adenovirus serotype 5 [2–4]. However, the receptor for adenovirus 5 has been reported to be downregulated in advanced tumors [5]. In this context, capsid-modified viruses have been developed, leading to higher transduction rates [2, 6]. One such example with clinical proof-of-concept data is the Ad5/3 chimera, which features the serotype 3 fiber knob on an otherwise Ad5 capsid [2, 7–12]. This approach avoids the problem of the Ad5 receptor by using the Ad3 receptor, proposed as desmoglein 2, which is highly expressed in advanced tumors [13].

In clinical trials, the route of delivery is a key factor determining practicality on one hand, and efficacy on the other. The majority of oncolytic virus trials have preferred to inject viruses directly into the tumor [14–18]. The advantage of this approach is that it maximizes delivery to the target, thereby reducing its interaction with other cells. On the other hand, restricted interstitial virus spread often limits infection beyond the needle track [19]. Since not all tumors are amenable to intratumoral injection, the treatment of disseminated cancers would benefit from systemic delivery by intravenous administration [20–22]. However, in this approach, most of the injected virus is lost to non-permissive tissues, and only a small proportion ends up in target tissues, where it may amplify, when an oncolytic platform is used. The advantage of intravenous delivery is that it allows system-wide viral distribution potentially into all tumors. In both humans and animal models, however, even intratumoral delivery leads to emphatic adenoviral presence in the blood [23]. This is caused by replication in tumor cells and subsequent shedding into the systemic compartments. Thus, both local and intravenous delivery have a systemic component.

There are several barriers that can affect the systemic delivery of a virus. Our immune system has developed mechanisms to prevent the spread of microorganisms, which do not differentiate between therapeutic viruses and pathogens. Systemic delivery of virus exposes it to various circulating factors, such as antibodies, that can block infectivity directly, or support viral clearance through Fc receptors found on phagocytes [24]. Other mechanisms include the complement system, other immune cells, and non-specific binding with the proteins in the serum [25]. Another important barrier that can reduce the bioavailability of a therapeutic virus is the organs, for example, spleen, liver, and lungs, which have resident macrophages. Their role is to scavenge pathogens in the blood, and they thus play a vital role in clearing circulating virus from the blood stream [25].

In virus-naïve individuals, these mechanisms are part of the innate immune system. However, in individuals with previous exposure, neutralization of the virus is much greater due to the involvement of the adaptive immune system. This represents an additional hurdle for advanced tumors, which requires repeated virus dosing for enhanced efficacy. In addition to these barriers, physical barriers in the tumor microenvironment and extracellular matrix limit virus extravasation [26].

Clinical data indicates that despite the presence of all these barriers, some but not all oncolytic viruses are able to reach tumors through blood [22, 27–29]. It has been a conundrum of how the viruses achieve this. In the present study, we have focused on documenting the interaction of chimeric adenovirus Ad5/3 with lymphocytes and erythrocytes, which we hypothesized might act as possible carriers for adenoviruses in the context of systemic administration.

## Materials and methods

### Cell lines

Human lung adenocarcinoma cancer A549 cell line was obtained from American Type Culture Collection (ATCC; LGS standards, USA). Prostate cancer PC-3MM2 cell line was gifted by Isaiah J. Fidler, M. D. Anderson Cancer Center. Both cell lines were free from mycoplasma contamination. A549 cells were maintained in Dulbecco’s modified Eagle’s medium (DMEM) and PC-3MM2 cells were maintained in RPMI. Both cell lines were supplemented with 5 or 10% fetal bovine serum (FBS), 1% l-Glutamine, and 1% Pen/Strep solution grown at 37°C with 5% CO_2._

### Viruses

Viruses Ad5/3-E2F-d24 [30], Ad5/3-E2F-d24-hTNFa-IRES-hIL2 (also known as TILT-123) [31] and Ad5/3-Luc1 [32] have been described previously. Briefly, replication-incompetent adenovirus Ad5/3-Luc1 features a knob domain from serotype 3 and contains firefly luciferase (Luc1) in a deleted E1 region. Replication-competent adenovirus Ad5/3-E2F-d24 was constructed by inserting E2F promoter in front of the adenoviral E1A gene that contains d24 deletion, so that the resulting E1A protein is unable to bind retinoblastoma (Rb) protein in cells. TILT-123 has a backbone similar to Ad5/3-E2F-d24 with transgenes placed into the E3 region under a replication-activated promoter.

### Preparation of blood cells

Human erythrocytes and lymphocytes were isolated from buffy coats of healthy donors obtained from the Finnish Red Cross Blood Service (Helsinki, Finland). Human erythrocytes and peripheral blood mononuclear cells (PBMCs) were isolated through density gradient separation, using Lymphoprep (StemCell technologie, Cambridge, UK; 07851). Since erythrocytes sediment through gradient medium, cells were collected from the pellet and washed twice with PBS. For short term storage, cells were treated with 10 % citrate-phosphate-dextrose (CPD, Sigma-Aldrich, USA; C7165) and stored at 4 °C.

PBMCs were carefully collected from the interface of plasma and gradient medium. Cells were washed twice with PBS and then treated with Ammonium-Chloride-Potassium ACK lysis buffer (Sigma, St Louis, MO. A10492-01) to lyse red blood cells. To isolate lymphocytes (CD14- cells) from fresh PBMCs, CD14+ magnetic beads were used according to the manufacturer instructions (Miltenyi Biotec, Sweden; 130–050–210).

### Quantification of Ad5/3 binding with human erythrocytes and lymphocytes

To study the binding at different concentration, TILT-123 was incubated with erythrocytes at 0.0036, 0.036, or 0.36 VP/cell and with lymphocytes at 1, 10 and 100 VP/cell in 1 ml PBS for 30 min at 37°C with continuous shaking. We have selected the ratios between cells and viruses based on a literature review [1, 33, 34]. For lymphocyte in vitro experiments, the ratio of 10 VP/cell is similar to the ratio used in studies with PBMCs [34]. For erythrocyte in vitro experiments, 1.8 × 10^8^ VP of Ad5/3 was incubated with 5 × 10^9^ erythrocytes (0.36 Ad particles per erythrocyte). The ratio is the same as the ratio used in [1, 33], and it resembles a clinically relevant dose (for 5 L of blood, it would correspond to an intravenous dose of 1 × 10^12^ VP, which is similar to a dose used in humans [35].

For binding experiments, Adenoviruses Ad5/3-Luc1, Ad5/3-E2F-d24, and TILT-123 were incubated with erythrocytes at 0.036 VP/cell [1] and with lymphocytes at 10 VP/cell [34] in 1 ml of PBS at 37°C with continuous shaking. After 30 min, samples were centrifuged for 10 min at 2000 g and cellular fraction and supernatant were collected. Cellular fraction was washed five times with PBS and each time a sample from pellet and supernatant was collected. DNA was extracted from collected samples using QIamp DNA kit (Qiagen, USA; 51304) to quantify the viral genome through quantitative polymerase chain reaction qPCR [36]. Viral copy number was normalized against the amount of genomic DNA in the sample, determined by the expression level of human β-actin.

### Adenovirus transduction and tumor cell killing in vitro

10,000 A549 cells per well were plated on 96-well plates 24 h before MTS cytotoxicity assay or Luciferase transduction assay. Erythrocytes and lymphocytes were incubated with TILT-123 or Ad5/3-Luc1 at the above-mentioned conditions. Following 30 min of incubation, samples were centrifuged and pellet was resuspended to the same volume *i.e.* 1 ml of assay medium; then 1:1.7, 1:2.7, and 1:6.7 dilutions of the cell–adenovirus mixture was added to A549 monolayers. For one group, the cell–adenovirus mixture was washed three times with PBS at 2000 g for 10 min and then 1:2.7 dilution of the pellet resuspended in 1 ml of assay medium was added to A549 monolayer. The main aim for the dilutions used in these experiments was to study the effect of virus-cell mix ranging from concentrated to unconcentrated. Erythrocytes alone and lymphocytes alone on A549 cells and erythrocytes with viruses and lymphocytes with viruses without A549 cells were used as negative controls. A549 cells infected with viruses in different concentrations (0.1–100 VP/cell) served as positive controls.

For the luciferase assay, infection medium was removed after 48 h and cells were incubated with lysis buffer (Promega, USA; A8261) at room temperature for 20 min and freeze-thawed once. The cell lysate was centrifuged and luciferase assay reagent (Promega, USA; E1500) was used to measure luciferase activity of the supernatant with a luminometer (Hidex). For cytotoxic assay, cell viability was determined with MTS (3-(4,5-dimethylthiazol-2-yl)-5-(3-carboxymethoxyphenyl)-2-(4-sulfophenyl)-2H-tetrazolium, inner salt)) assay (Cell titer 96 Aqueous One Solution Cell Proliferation Assay, Promega, USA; G3582) on day 3.

For the migration assay, 50,000 A549 cells were seeded on the lower chamber of a 24-well Transwell plate (Corning Costar; 3415) 24 h before the experiment. Erythrocytes and lymphocytes were incubated with TILT-123 at the above-mentioned conditions. Following 30 min of incubation, samples were centrifuged, and the pellet was resuspended in the same volume *i.e.* 1 ml of assay medium. 300 µl of this mix was added on the transwell surface (3 µm pore size) and incubated for 4 h at 37°C. As a negative control, erythrocytes alone and lymphocytes alone on A549 cells and erythrocytes with viruses and lymphocytes with viruses without A549 cells were used (not shown). A549 cells infected with TILT-123 alone at 0.1 VP/cell, 1 VP/cell, 10 VP/cell, and 100 VP/cell were used as positive controls. On day 3, MTS assay (Cell titer 96 Aqueous One Solution Cell Proliferation Assay, Promega, USA; G3582) was used to determine the cell viability.

### Electron microscopy

TILT-123 was incubated with freshly isolated human lymphocytes and with human erythrocytes in 1 ml of Phosphate Buffered Saline (PBS) at 37°C for 30 min. After incubation, the cellular fraction obtained through centrifugation was fixed in 2.5 and 5% glutaraldehyde respectively, according to the protocol used at the University of Helsinki Electron Microscopy Unit. Scanning electron microscopy (SEM) samples were prepared and analyzed in Electron Microscopy Unit at the University of Helsinki. Samples for transmission electron microscopy (TEM) were analyzed in Advance Microscopy Unit (AMU) at the University of Helsinki, Finland.

### Animal studies

All the animal protocols were approved by the Provincial Government of Southern Finland and the experimental animal committee of the University of Helsinki. Five-week-old immunodeficient NMRI (female) mice were implanted subcutaneously with 2 × 10e6 human prostate cancer PC-3MM2 cells or 5 × 10e6 human lung adenocarcinoma A549 cells. All the mice with established tumors were included. When tumors became injectable, mice were randomized (according to the tumor size) into eight groups of 5–7 mice. Mice carrying PC-3MM2 tumors were treated intravenously with TILT-123 previously incubated with human lymphocytes or erythrocytes 500 virus particles (VP)/cell. Positive control and negative mock control received 1.5 × 10e10 VP/100 µl of TILT-123 and PBS, respectively. In the animal experiment with A549 tumors, we increased the virus doses to 2 × 10e9 VP/100 µl as experimental dose and 2 × 10e10 VP/100 µl of TILT123 as a positive control. Mice received intravenously 667 VP/cell (2 × 10e9 VP in total) of TILT-123 previously incubated with human lymphocytes or erythrocytes.

To evaluate the effect of neutralizing antibodies (NAbs) on the efficacy of adenovirus-cell complexes, we generated neutralizing antibodies by immunizing immunocompetent mice three times on days 0, 3, and 6. Blood was collected on day 23 to separate serum. NAb titer in serum was confirmed with NAb assay [37] and the titer that blocked more than 50% of the virus was used. PC-3MM2 and A549 xenograft-bearing immunodeficient mice received the same treatments as before, but the virus or virus-cell complexes were first incubated with heat-inactivated antiserum for at least 30 min.

After day 3, mice were euthanized and tumors were collected and snap-frozen to detect adenovirus Ad5/3 genome through qPCR and to detect transgene expression (human tumor necrosis factor-alpha (TNF-α) and Interleukin 2 (IL-2)) through flex set bead arrays (Becton Dickinson (BD) Cytometric Bead Array human flex set; BD biosciences, USA; 558273 and 558270).

### Statistics

We used Shapiro–Wilk test to assess the normality of the outcome data, and Levene’s test to test equality of variances. According to these test data was non-normal and the variances between the groups were different, so non-parametric Kruskal–Wallis test was used to compare groups. If the result is statistically significant (*p*-value < 0.05), then we carried out the post hoc analyses to compare groups pairwise using Dunn’s test. *P*-values of post hoc (p-h) analyses were adjusted using Holm multiple testing correction method. Statistical analyses and figures were made using Graph pad Prism 6 (graph Pad Prism Software Inc., San Diego, CA) and R statistical software (R Core Team (2019).

## Results

### Ad5/3 adenoviruses are able to bind to human lymphocytes and erythrocytes

The binding of adenovirus Ad5/3 with lymphocytes and erythrocytes was studied at different VP/cell. Ad5/3 virus was incubated with lymphocytes at 1, 10, and 100 VP/cell and with erythrocytes at 0.0036, 0.036, and 0.36 VP/cell for 30 min, followed by centrifugation and viral DNA quantification in the cellular fraction (Supplementary Fig. 1). We saw virus binding with lymphocytes and erythrocytes at different VPs: the higher the input the higher the amount of virus bound.

**Fig. 1:**
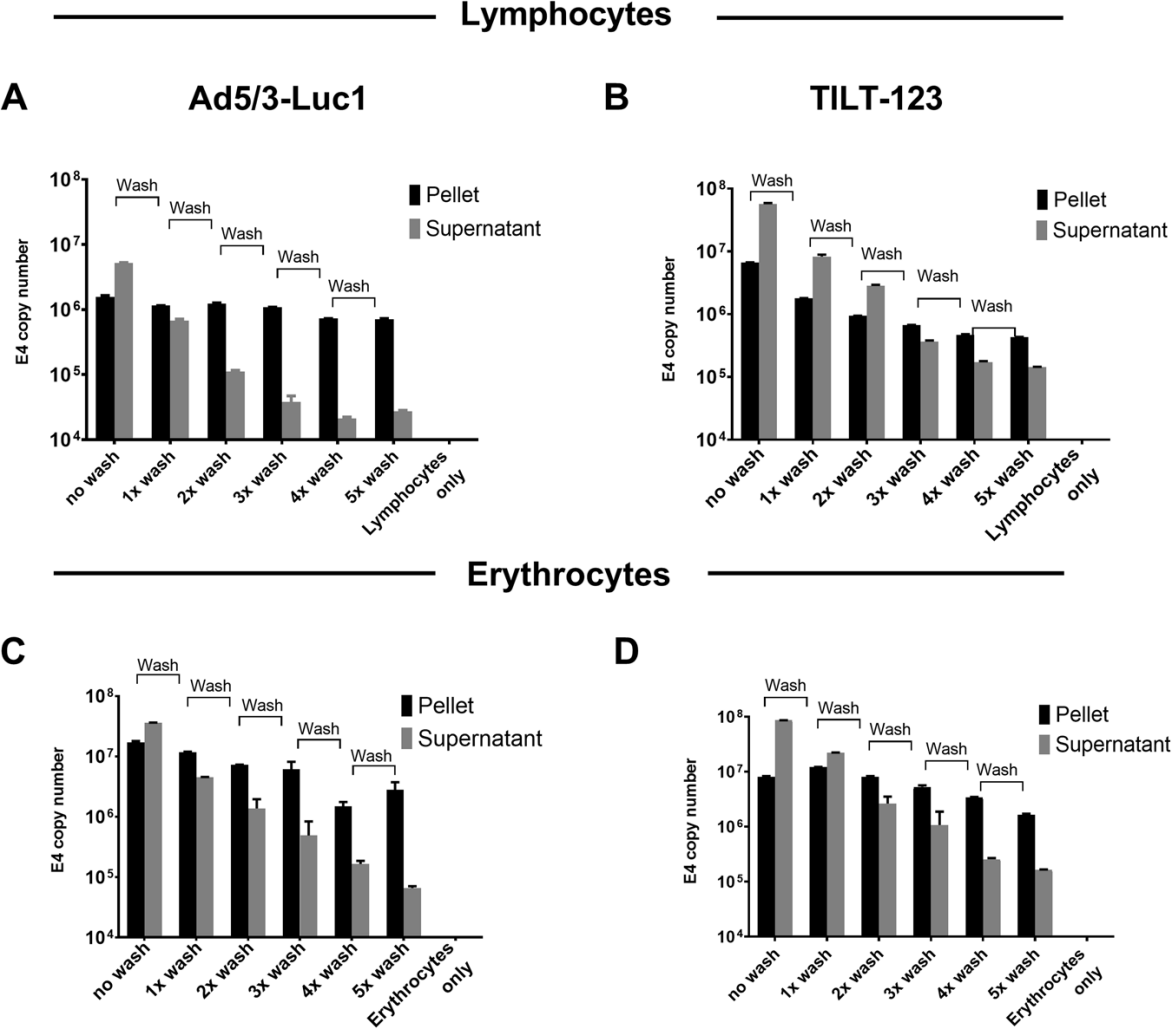
Association of adenovirus particles with lymphocytes and erythrocytes. Adenovirus Ad5/3-Luc1 (**a**, **c**) and TILT-123 (**b**, **d**) were incubated with human lymphocytes at 10 VP/cell (3 × 10e7 VP/ml with 3 × 10e6 cells/ml) (**a**, **b**) and with erythrocytes at 0.036 VP/cell (5 × 10e9 VP/ml and 1.8 × 10e8 cells/ml) (**c**, **d**) at 37°C. After 30 min incubation, cellular fraction and supernatant were isolated through centrifugation at 2000 × *g* for 10 min. Cellular fraction was washed five times and after each wash a sample from the supernatant and pellet was collected and analyzed through qPCR. Viral copy number was normalized against amount of genomic DNA in the sample, determined by the expression level of human β-actin. Data are presented as mean + SEM. Adenovirus particles associated with cell fraction (black bar) and supernatant (Sup, gray bars). No wash; first centrifugation after incubation, 1x wash; washed with PBS once, 2x wash; washed twice, 3x wash; 4x wash; 5x wash; washed three, four and five times, respectively.

In order to determine the binding of Ad5/3 adenoviruses with lymphocytes (at 10 VP/cell) (Fig. 1a, b) and with erythrocytes (at 0.036 VP/cell) (Fig. 1c, d), supernatants were collected after 30-min incubation by centrifugation and virus DNA was quantified. The cellular fraction was washed five times and samples were collected after each wash. Ad5/3 adenoviruses were able to maintain the quite consistent binding with the selected blood cell types after each wash. Although the supernatant was removed at every wash, unbound virus could be detected in the supernatant after every round of centrifugation and wash. Thus, we saw a continuous association of the virus with erythrocytes and lymphocytes, confirming that a proportion of the virus persists in the cellular fraction.

### Ad5/3 binding with the lymphocytes and erythrocytes is reversible and does not inhibit virus oncolytic capacity

Next, we evaluated whether the binding of Ad5/3 with human lymphocytes and erythrocytes inhibits adenovirus transduction. We used replication-deficient Ad5/3-Luc1 to detect if the virus is able to transduce cancer cells when delivered with lymphocytes or erythrocytes, which would lead to luciferase expression. Similarly, replication-competent TILT-123 was used to detect cell-killing efficacy with MTS assay [31].

**Fig. 2:**
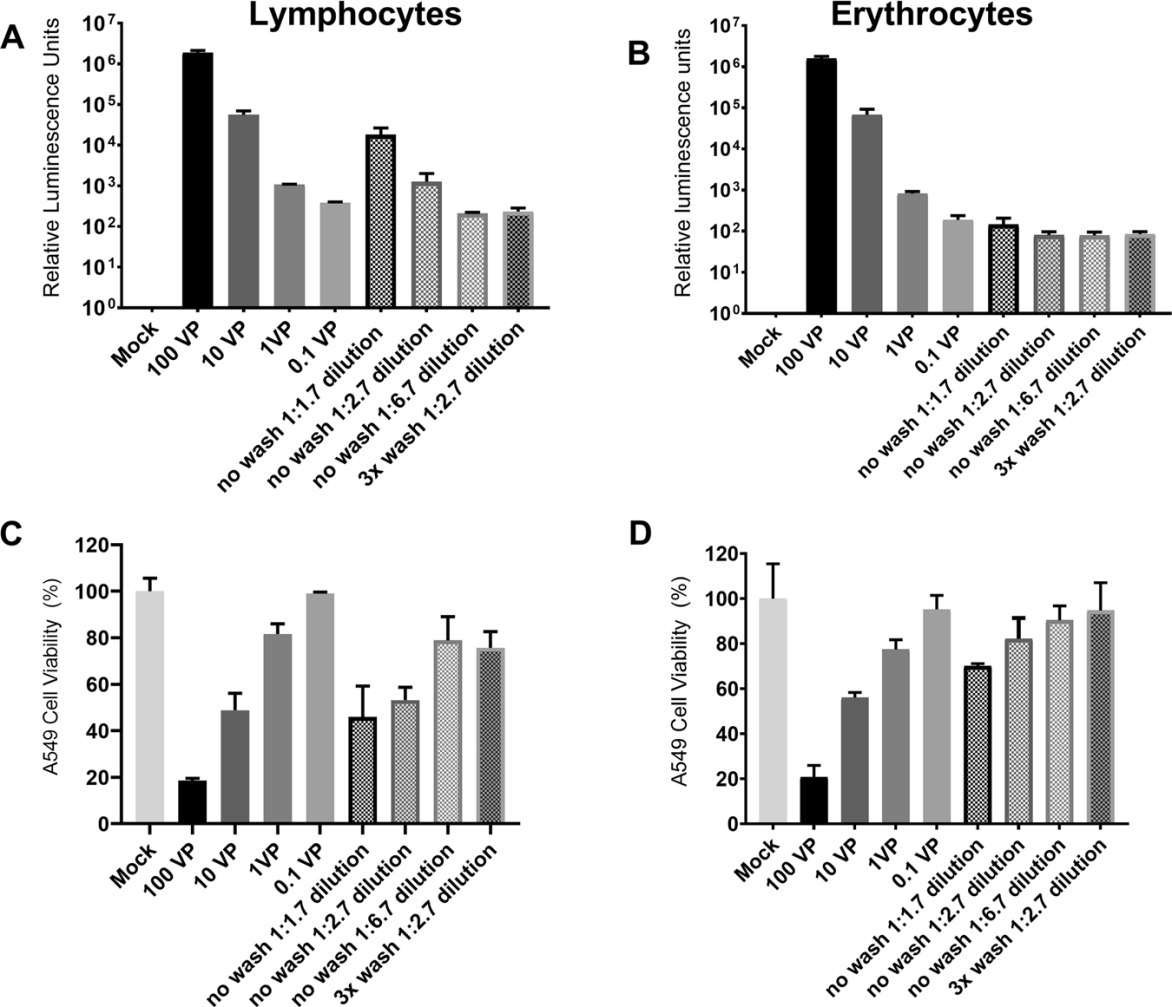
Adenovirus transduction and tumor cell killing potential after interaction with lymphocytes and erythrocytes. Ad5/3-Luc1 (**a**, **b**) and TILT-123 (**c**, **d**) were incubated at 37°C for 30 min with human lymphocytes (**a**, **c**) at 10 VP/cell or with human erythrocytes (**b**, **d**) at 0.036 VP/cell. After incubation, samples were centrifuged, resuspended and then 1:1.7, 1:2.7, and 1.6.7 dilutions of cell–adenovirus mixture was added to A549 monolayer. For one group, cell–adenovirus mixture was washed three times with PBS at 2000 × *g* for 10 min and then 1:2.7 dilution of cell suspension was added to A549 monolayer. Luciferase expression was measured after 48 h (**a**, **b**). Tumor-killing ability of TILT-123 as such (0.1–100 VP/cell) or when delivered with lymphocytes (**c**) or erythrocytes (**d**) was analyzed on day 3 with cytotoxicity (MTS) assay on A549 cells. Data are presented as mean + SEM. No wash; first centrifugation after incubation, 3x wash; washed with PBS three times.

Cells infected with Ad5/3-Luc1 showed clear transduction regardless of the presence of lymphocytes or erythrocytes (Fig. 2a, b). When a 1:1.7 dilution of the lymphocytes plus Ad5/3-Luc1 mixture was incubated with A549 carcinoma cells, we observed a luciferase expression level comparable to 10 VP/cell. When 1:2.7 and 1:6.7 dilutions of the lymphocytes plus Ad5/3-Luc1 mixture were incubated with A549 cells, the luciferase expression levels were comparable to 1 VP/cell and 0.1 VP/cell, respectively (Fig. 2a). When the cell-virus mixture was washed three times before plating it on A549 cells, the luciferase expression was similar to 0.1 VP/cell. With erythrocytes, all conditions led to similar levels of transgene expression, comparable to 0.1 VP/cell, including the group where cells–adenovirus mixture was washed three times before infection (Fig. 2b). A luminescent signal was not observed from negative control samples *i.e.* erythrocytes or lymphocytes incubated with Ad5/3-Luc1 in the absence of A549 cells (Supplementary Fig. 2). These results showed that adenovirus Ad5/3 binding with either lymphocytes or erythrocytes is reversible and the released virus is able to infect cells.

The oncolytic potency of cell-bound adenovirus was studied with the replication-competent TILT-123 virus (Fig. 2c, d). We observed maximum cell killing in our positive control (*i.e.* 100 VP/cell). When 10 VP/cell of TILT-123 was used, we found comparable cell killing to 1:1.7 and 1:2.7 dilutions of lymphocytes mixed with TILT-123 till day 3 (Fig. 2c). When we used 1:6.7 dilutions of the lymphocytes plus TILT-123 mixture or when the 1:2.7 diluted cell-virus mixture was washed three times, cell killing was comparable to 1 VP/cell (Fig. 2c). Thus, indicating that adenovirus binding with these cell types does not inhibit the oncolytic potency of the virus. With erythrocytes, 1:1.7 and 1:2.7 dilution of the cell-virus mixed were comparable to 10 VP/cell and 1 VP/cell respectively. When 1:6.7 dilutions of the erythrocytes plus TILT-123 mixture or when the cell–virus mixture was washed three times, then 1:2.7 dilution of it was used, cell killing was comparable to 0.1 VP/cell (Fig. 2d). Thus, we showed that the virus is still fully functional even after binding with either lymphocytes or erythrocytes.

The cell-killing ability of cell-bound adenoviruses was further studied through migration assay. We observed that the potency of cell-bound adenovirus TILT-123 is comparable to unbound adenovirus at 1 VP/cell. The results indicate that some of the viruses was released from erythrocytes and lymphocytes, migrated through the transwell, and killed A549 cells. Thus, it further confirmed that adenovirus, in this case, TILT-123, retain oncolytic ability even after binding to (and subsequent release from) lymphocytes or erythrocytes (Supplementary Fig. 3).

### Adenovirus Ad5/3 binds to the surface of the lymphocytes and erythrocytes

**Fig. 3:**
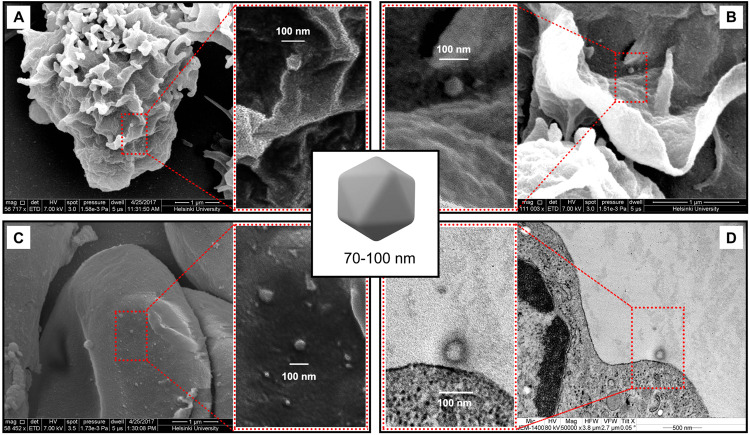
Electron microscopy view of Ad5/3 adenovirus with lymphocytes and erythrocytes. Scanning electron microscopy (SEM) images of TILT-123 that was incubated at 37°C for 30 min with human lymphocytes (**a**, **b**) and with human erythrocytes (**c**). (**d)** Transmission electron microscopy (TEM) view of Ad5/3 adenovirus with lymphocytes. Arrow indicates binding of virus at the surface of the cells.

To visualize Ad5/3 virus binding with blood cells, we incubated TILT-123 with lymphocytes and erythrocytes and envisaged the samples through electron microscopy (SEM and TEM). Adenovirus is a non-enveloped virus and has icosahedral capsid (~90 nm) [38]. SEM images showed that Ad5/3 chimeric adenovirus was able to bind on the surface of lymphocytes (Fig. 3a, b) and erythrocytes (Fig. 3c). Thus, we were able not only to detect and identify virus through SEM but also to confirm its surface association with human lymphocytes and erythrocytes.

According to previous knowledge, erythrocytes are not able to internalize adenoviruses [39], but this has not been well studied for lymphocytes. To confirm the binding of adenovirus Ad5/3 to lymphocytes, we analyzed lymphocyte-adenovirus mixture through TEM. Interestingly, we did not find any internalized adenoviruses, but we have found TILT-123 bound to the surface of a lymphocyte (Fig. 3d). Thus, the results further confirm our finding that Ad5/3 adenovirus has a surface association with the selected cell types.

### Adenovirus Ad5/3 binding with erythrocytes and lymphocytes is reversible and does not inhibit tumor transduction in vivo

Systemic delivery of oncolytic adenoviruses is an attractive approach, because it simultaneously provides the possibility to treat the primary tumor and metastatic tumors [40]. Therefore, we decided to study in vivo, whether erythrocytes and lymphocytes could effectively deliver Ad5/3 into tumors and whether binding to these cells could protect the virus from neutralization. Immunodeficient mice bearing subcutaneous human prostate tumors (PC-3MM2) were administered intravenously with TILT-123, either with virus alone (1.5 × 10e10 VP/100 µl) as positive controls or 1.5 × 10e9 VP/100 µl as experimental control) or bound to erythrocytes or lymphocytes 500 VP/cell.

**Fig. 4:**
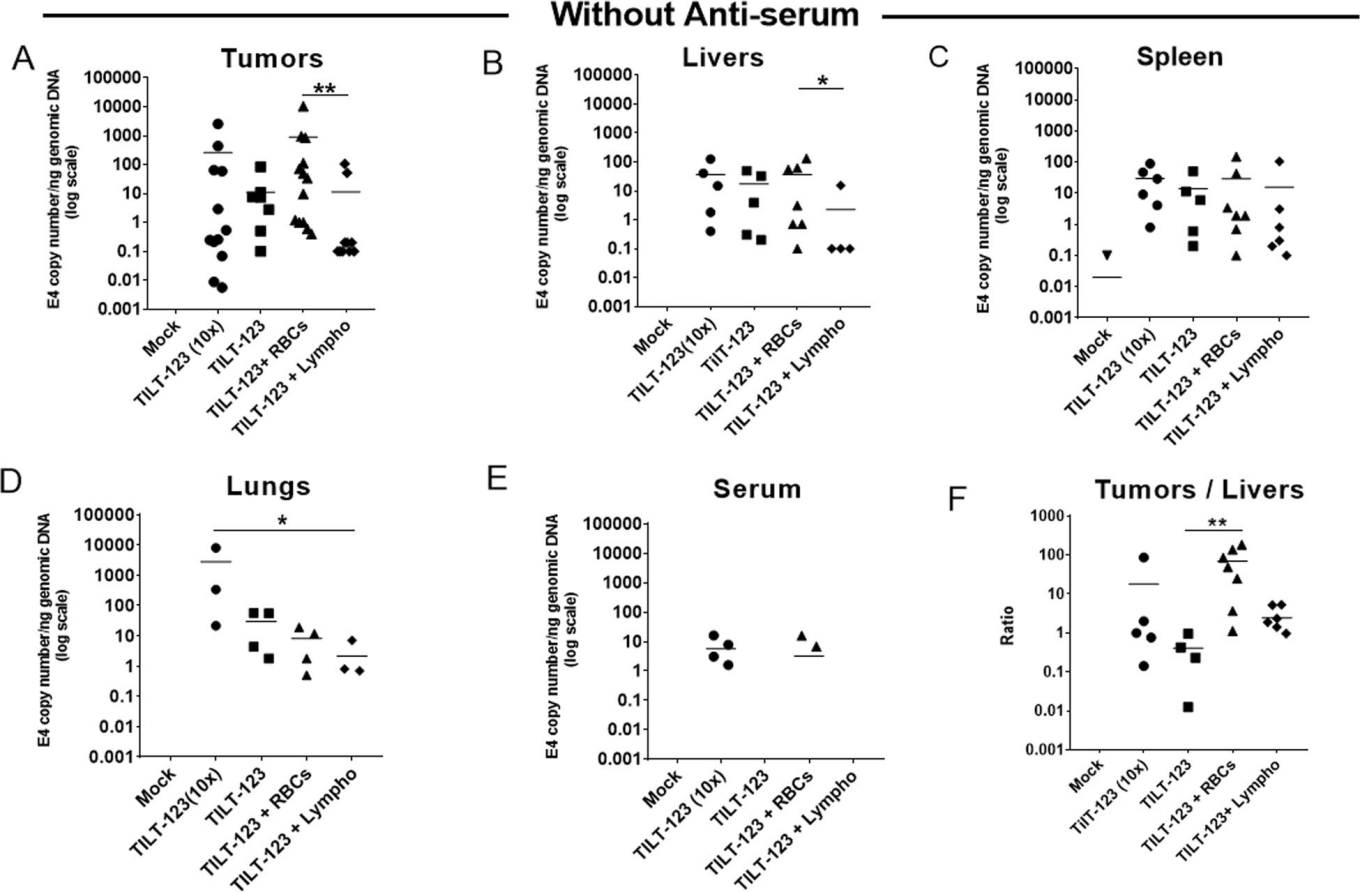
Adenovirus systemic transduction of prostate tumors in the presence or absence of lymphocytes and erythrocytes. Immunodeficient NMRI mice bearing subcutaneous PC3MM2 prostate tumors were injected intravenously with TILT-123 previously incubated with or without human lymphocytes or erythrocytes (500 VP/cell). Positive control and negative mock control received ten times (10×) more virus (1.5 × 10e10 VP) or PBS, respectively. After day 3 post treatments, the mice were euthanized and tumors (**a**), livers (**b**), Spleen (**c**), lungs (**d**), and serum (**e**) were collected and snap-frozen to detect adenovirus Ad5/3 genomes through qPCR. Viral copy number was normalized against amount of genomic DNA in the sample, determined by the expression level of human β-actin (for tumors) and mouse β-actin (for murine samples). Copy number ratios in tumors vs. livers were calculated to evaluate the distribution of the virus (**f**). Data are presented as mean. RBC: erythrocytes; Lympho: lymphocytes. **P < 0.01, *P < 0.05 by two tailed post hoc Dunn’s test adjusted by Holm multiple testing correction method.

After intravenous injection, all groups (*i.e*. virus alone groups or virus bound with cells) showed presences of adenovirus DNA in tumors as measured by qPCR. Virus delivery with erythrocytes or lymphocytes did not improve tumor transduction. However, the presence of TILT-123 DNA in tumors was slightly increased when delivered with erythrocytes, as compared with lymphocytes (Fig. 4a, p–h; *p* = 0.00575). Thus, we have shown that binding of the virus with erythrocytes or lymphocytes did not impede tumor transduction. In addition, groups treated with intravenously injected TILT-123, with or without binding to erythrocytes and lymphocytes, viral DNA was efficiently present in liver, spleen, and lungs (Fig. 4b–d). When the virus was delivered with lymphocytes, we found less viral DNA in liver than when delivered with erythrocytes (p–h; *p* = 0.0477). Viral DNA in lungs was statistically significantly lower when TILT-123 was bound to lymphocytes, compared with control condition (10x) (Fig. 4d, p–h; *p* = 0.0374). We detected extended blood persistence of the virus in the positive control group (10x more virus was delivered) as compared to other groups (Fig. 4e). Thus, it corroborates that human erythrocytes and lymphocytes did not prevent adenovirus transduction in tumor and organs.

Importantly, analysis of tumor-to-liver ratios showed that adenovirus was able to transduce tumors over livers better when bound with lymphocytes (mean 2.44) and significantly better, when bound with erythrocytes (p–h; *p* = 0.00798; mean 68.93), over the virus that was injected alone (mean 0.4). Especially, when TILT-123 was bound with erythrocytes, preference for tumor transduction was even more prominent than in the positive control group (10× more virus intravenously, mean 18) (Fig. 4f). The liver is the organ responsible for most virus uptake and therefore that is a relevant comparison [41].

**Fig. 5:**
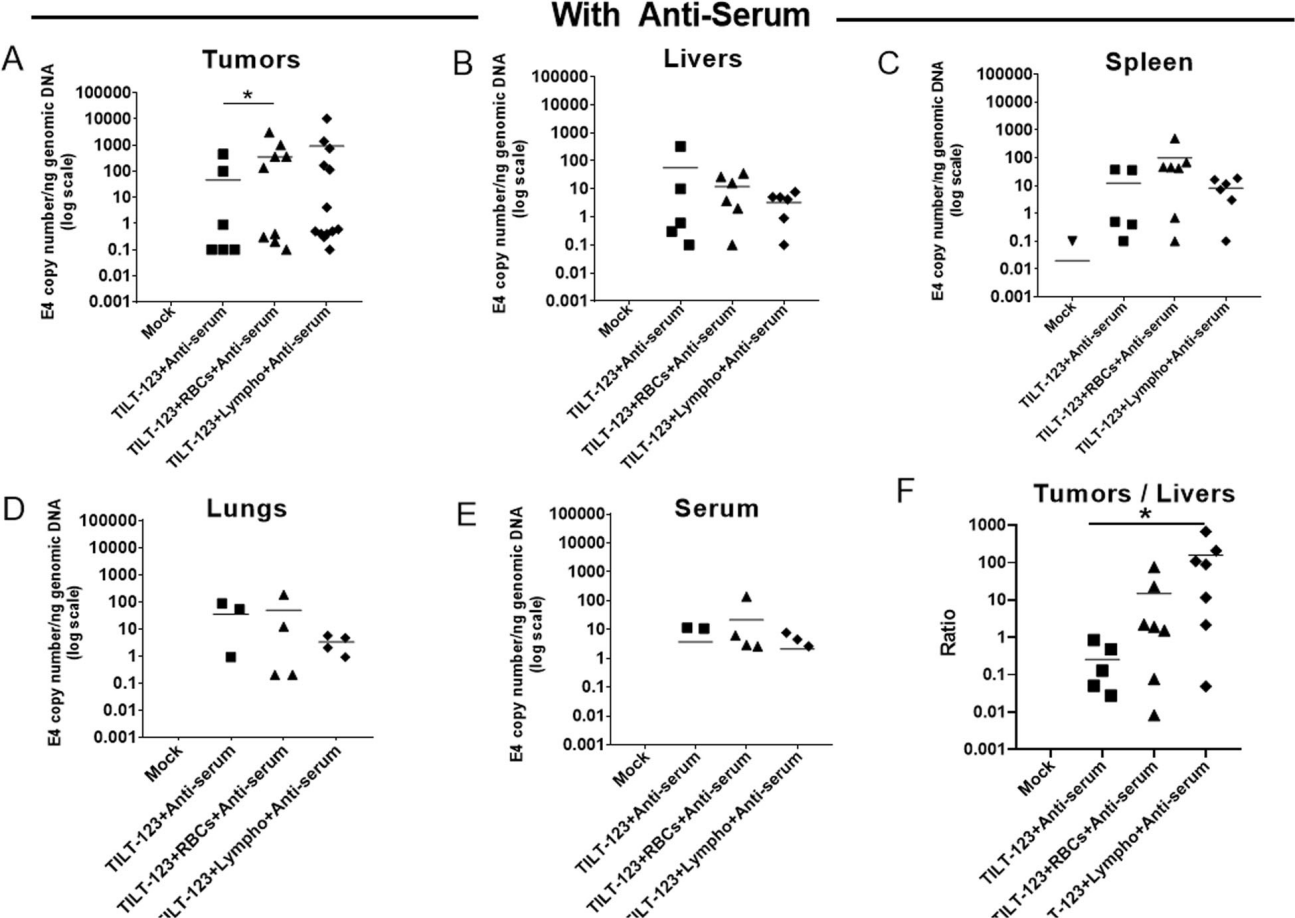
Adenovirus systemic transduction of prostate tumors in the presence or absence of lymphocytes and erythrocytes along with Ad5/3-specific antiserum. Immunodeficient NMRI mice bearing subcutaneous PC3MM2 prostate tumors were injected intravenously with TILT-123 previously incubated with or without human lymphocytes or erythrocytes (500 VP/cell) in the presence of Ad5/3-specific antiserum. Negative mock control received PBS. After 3 days post treatments, the mice were euthanized and tumors (**a**), livers (**b**), spleen (**c**), lungs (**d**), and serum (**e**) were collected and snap-frozen to detect adenovirus Ad5/3 genomes through qPCR. Viral copy number was normalized against amount of genomic DNA in the sample, determined by the expression level of human β-actin (for tumors) and mouse β-actin (for murine samples). Copy number ratios in tumors vs. livers were calculated to evaluate the distribution of the virus (**f**). Data are presented as mean. RBC: erythrocytes; Lympho: lymphocytes. *P < 0.05 by two tailed post hoc Dunn’s test adjusted by Holm multiple testing correction method.

TILT-123 bound to erythrocytes (p–h; *p* = 0.0352) and lymphocytes despite neutralizing antiserum, and showed the enhanced presence of virus DNA in tumors as compared to when the virus alone was mixed with antiserum (Fig. 5a). Interestingly, when the virus alone was mixed with antiserum, we saw marginally more presence of viral DNA in the liver (Fig. 5b).

Thus, we have shown that even in the presence of neutralizing antibodies, cells were able to deliver Ad5/3 virus. Of note, when adenovirus Ad5/3 was bound with cells, more tumor transduction was seen as compared to the liver even in the presence of neutralizing antibodies (mean values; TILT-123 plus antiserum 0.26; TILT-123 plus erythrocytes plus antiserum 14.85; TILT-123 plus lymphocytes plus antiserum 156.68; post hoc test between TILT-123 plus antiserum and TILT-123 plus lymphocytes plus antiserum (*p* = 0.0134)) (Fig. 5f).

**Fig. 6:**
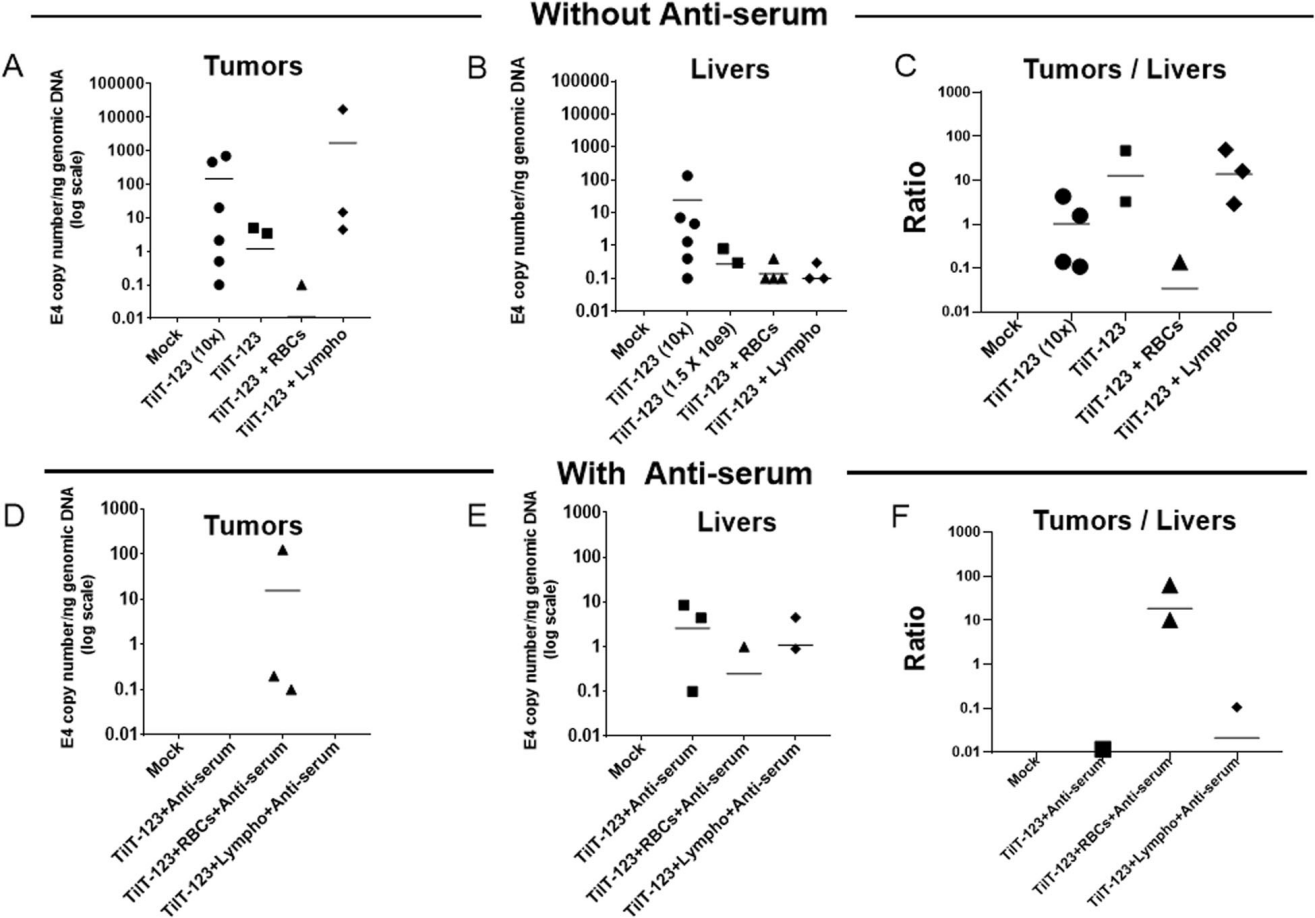
Adenovirus systemic transduction of lung tumors in the presence or absence of lymphocytes and erythrocytes. Immunodeficient NMRI mice bearing subcutaneous A549 human lung adenocarcinoma tumors were injected intravenously with 2 × 10e9 VP/100 µl of TILT-123 alone or previously incubated with or without either human lymphocytes or erythrocytes (667 VP/cell) and with or without antiserum. Positive control and negative mock control received 2 × 10e10 VP/100 µl of TILT-123 and PBS, respectively. After 3 days, mice were euthanized. Tumors (**a**) (**d** with cell-virus mixed with antiserum) and livers (**b**) (**e** with cell-virus mixed with antiserum) were collected and snap-frozen to detect adenovirus Ad5/3 genome through qPCR. Copy number ratios in tumors vs. livers were calculated to evaluate the distribution of the virus (**c**, **f**). Viral copy number was normalized against amount of genomic DNA in the sample, determined by the expression level of human β-actin (for tumors) and mouse β-actin (for murine samples). Data are presented as mean. RBC: erythrocytes; Lympho: lymphocytes.

Every tumor is different with regard to tumor vasculature and tumor microenvironment, making them respond differently to oncolytic viral therapy [42]. When using mice (or any animal model), every individual mouse differs, for example, in the speed of tumor uptake, tumor size, and formation of the vasculature. All these play a role in tumor transduction variation. The same phenomenon is also seen in humans. Therefore, we repeated all the above-mentioned experiments in a similar way, but using a different tumor model: human lung adenocarcinoma A549 tumors. In this experiment, we found more Ad5/3 virus DNA in tumors, livers, and spleen from the positive control group receiving ten times more virus intravenously than the other groups. Treatment with the experimental dose of TILT-123 alone and TILT-123 bound with lymphocytes had comparable delivery efficacy of virus to tumors (Fig. 6a) and to a lesser extent to livers (Fig. 6b) and spleen (Supplementary Fig. 4a). In this experiment, we did not find the presence of the virus in blood serum except for the one mouse from the positive control group (Supplementary Fig. 4b).

The mean of tumor-to-liver ratios were 1.017 in the positive control (10X) group, 12.78 in experimental virus dose group, 0.034 in virus bound with erythrocyte group and 13.75 in virus bound with lymphocyte group (Fig. 6c). However, no significant differences were observed between the groups. For the erythrocytes group, only one tumor of one mouse has a slightly positive E4 copy number/ng genomic DNA value of 0.1 (Fig. 6a). In the tumor samples, we detected the expression of IL-2 and TNF-α, which is associated with virus replication, confirming delivery of functional virus to the tumor (Supplementary Fig. 5a, b).

When we added neutralizing antiserum, we found most of the virus DNA in the group that received virus bound to erythrocytes plus antiserum as compared to other groups (Fig. 6d). In addition, there was less virus DNA found in liver and spleen in this group (Fig. 6e and Supplementary Fig. 4c). We did not detect virus in the blood serum in any group (Supplementary Fig. 4d). In this experiment, anti-serum had a greater neutralizing effect on the virus alone, which led to more virus in liver as compared to a tumor, as liver uptake (by e.g. Kupffer cells) is not dependent on interaction with the primary receptor of virus [43, 44]. In case of virus plus lymphocytes and antiserum, we saw a pronounced neutralizing effect (Fig. 6d–f and Supplementary Fig. 4c).

The mean of tumor-to-liver ratios were 0.0024 in the virus plus antiserum group, 18.49 in virus plus erythrocytes plus antiserum group, and 0.4 in virus plus lymphocytes plus antiserum group. We observed the expression of IL-2 and TNF-a in the tumor samples, demonstrating virus replication (Supplementary Fig. 5c, d).

## Discussion

Oncolytic adenoviruses have been widely used and have a safe clinical profile [22, 45, 46]. Moreover, there is human data showing that intravenous delivery of oncolytic adenoviruses can result in the transduction of metastases through the blood stream. However, the mechanism has been unclear [45, 47–50].

Upon intravenous injection, adenovirus encounters many barriers that may hinder transduction. For example, liver sinusoidal endothelial cells and Kupffer cells sequester virus particles circulating in the blood [51]. When adenovirus is administered into the bloodstream, it gets exposed to various circulating factors, especially blood cells and proteins of the plasma. Interestingly, antibodies that either previously existed or develop after the exposure, also reduce the bioavailability of the virus, for example by blocking the capsid directly, or by clearance through phagocytes’ Fc receptors [46]. The effects of plasma components on virus neutralization have been reported [37, 52], but not much is known about the interactions between blood cells and adenovirus.

Thus, given all the obstacles present in the blood, it has been unknown how adenovirus is able to reach systemic metastases. Interestingly, it was recently documented that oncolytic reovirus binds with blood cells such as monocytes, which deliver the reovirus to tumors despite the presence of neutralizing antibodies [53]. We hypothesized that a similar phenomenon could be true also for adenoviruses. Specifically, Ad5/3 has shown improved tumor transduction and antitumor efficacy [31]. Therefore, we thought Ad5/3 virus would be able to bind with blood cells, and that this binding would be reversible. In blood, lymphocytes and erythrocytes are among the most abundant cell types so the focus here was on these subsets.

Of note, we found that the interaction between adenovirus Ad5/3 and blood cells does not affect the cell-killing ability of the virus. We saw that erythrocytes and lymphocytes may be able to protect surface-adhered adenovirus, and are also capable of delivering it into the tumor even in the presence of neutralizing antibodies.

Human adenovirus enters host cells after interacting with two different cell receptors. Initially, viral fiber capsid protein binds to a primary receptor, which differs depending upon the serotype [54]. The subsequent entry then occurs through the binding of viral penton base to cellular αv integrins [55, 56]. The second interaction is mediated through the penton base tripeptide Arg-Gly-Asp (RGD) sequence motif, which is known to be conserved in many serotypes [55]. Lack of either interaction impedes adenovirus entry. The primary interaction of adenovirus with cells is through the fiber knob, which in the case of adenovirus 5/3 is the serotype 3 knob, which binds to desmoglein 2 [57].

Adenoviruses are known to infect a wide range of epithelial cells. However, lymphocytes and erythrocytes are not susceptible to adenovirus infection. This is probably mainly due to the failure of adenovirus to be internalized [33, 58, 59]. For example, adenovirus’ inability to replicate in T cells is due to the fact that T lymphocytes express fiber receptors at low levels [58, 59]. Moreover, T lymphocytes also express limited levels of α_V_β integrins, which are critical for the internalization of adenovirus after primary attachment [58]. Importantly, human erythrocytes lack integrins and are, therefore, not permissive to internalization of adenoviruses [33].

It has been proposed that some viruses can hitchhike on the surface of blood cells [60]. Thus, cells may be able to deliver virus to tumor, explaining the fact that some oncolytic viruses have been shown to be able to infect distant tumors through the bloodstream despite humoral obstacles [29]. Possible mechanisms of cells delivering viruses to tumor include cellular synapses between tumor and blood cells, or viruses passively “falling off” cells into tumors [61]. The latter would appear feasible if there is an equilibrium or homeostasis between cell-bound and free virus in the liquids surrounding cells. Our results show that lymphocytes and erythrocytes bind with Ad5/3 adenoviruses quite firmly, but in a reversible and balance-seeking manner, so that even after five centrifugations and washes we could detect Ad5/3 in both the supernatant and attached to cells. Of key importance, we saw that this interaction was reversible and does not inactivate virus, as confirmed with different assays including luciferase, cytotoxicity, and migration assays. Further, we found that adenovirus Ad5/3 only interacts with the surface of the cells (instead of entering), using scanning and transmission electron microscopy.

Our results demonstrate the ability of adenovirus Ad5/3 to “hitchhike” on human blood cells in vivo, in two different tumor models. Interestingly, erythrocytes and lymphocytes changed the virus tropism towards tumor over the liver, resulting in higher tumor-to-liver ratios of virus genomes. Similarly, even in the presence of serum-containing neutralizing antibodies against 5/3 adenovirus, both cell types were able to deliver more virus to the tumors than normal organs, indicating that the cells protected virus from neutralization. For lymphocytes, a possible mechanism is their cytoplasmic projections that, to some extent, may prevent the direct interaction of the virus with neutralizing antibodies. For erythrocytes, a possible mechanism is that they can change their shape [62]. This may, to some extent, prevent the direct interaction of virus with neutralizing antibodies. Also, complete opsonisation may be prevented by binding to the cell surface. Importantly, we found that virus that had transduced tumor was functional and able to express its transgenes IL-2 and TNF-a. TILT-123 is a construct featuring replication-associated transgene expression and therefore transgene expression indicates virus replication [31].

The ability of adenoviruses to bind with erythrocytes is species specific and differs substantially between humans and animal models [63]. For the adenovirus to bind to erythrocytes and lymphocytes it needs the interaction of fiber protein with the adenovirus receptor, which in this case is desmoglein 2. Lack or low expression of these receptors leads to less binding of human adenovirus with murine erythrocytes or lymphocytes. Adenovirus interacts with murine and human blood cells differently [64]. It has been previously studied that human adenovirus serotype 5 interacts negligibly with freshly isolated murine blood cells (<0.1% binding). Moreover, a study with intravenous administration of adenovirus 5 shows that it does not bind with murine erythrocytes and over 99% of viral genomes in the murine bloodstream are free in the plasma [34]. Rojas et al. 2016 evaluated the persistence of the intravenously transferred human erythrocytes in the blood of nude mice. Even though human erythrocytes in mice were rapidly cleared from the blood circulation, kinetics of clearance of human erythrocytes was slower as compared with that of adenovirus’. Therefore, preincubation with human cells changes the biodistribution of the oncolytic virus [1]. It has been reported that human erythrocytes sequester adenovirus and, thus, impact infectivity [14, 33]. A study reported a high affinity between adenovirus serotype 5 and human erythrocytes, enabling Ad5 to adhere to the surface of human erythrocytes. As in our study with Ad5/3, ~98% of Ad5 was associated to the surface of erythrocytes [63]. However, in the context of an oncolytic virus, virus dissociating from cells at tumors can replicate, and therefore even a small proportion of virus releasing from cells could yield a therapeutic effect. Our finding that human 5/3 chimeric adenovirus is able to hitchhike on human erythrocytes to reach non-injected tumors is in accord with a previous report on Ad5, which suggested that this phenomenon can improve anti-tumor efficacy [39].

Importantly, our results reveal that when Ad5/3 is bound to erythrocytes, the tumor is favorably transduced over normal organs, even in the presence of anti-Ad5/3 antiserum, leading to increased tumor-to-liver ratios. Interestingly, we did not see pronounced neutralization by anti-Ad5/3 antiserum, when cells were present. Thus, apparently binding to human erythrocytes and lymphocytes was able to avoid neutralization of Ad5/3 by antibodies. It will be interesting to study if Ad5/35 or Ad11 behaves more like Ad5 or Ad5/3 in the context of the interactions studied here.

In summary, in this paper, we have discovered that the Ad5/3 chimeric adenovirus is able to transduce non-injected tumors through blood, even in the presence of neutralizing antibodies. It does so by binding reversibly to human erythrocytes and lymphocytes. When these cells squeeze into tumor capillaries [65], viruses can dissociate from cells, enter tumor cells, and start replicating.

## Supplementary information

Supplementary figure Legends

Supplementary figure 1

Supplementary figure 2

Supplementary figure 3

Supplementary figure 4

Supplementary figure 5
