# Supplementary material for: Ad5/3 is able to avoid neutralization by binding to erythrocytes and lymphocytes
Source: Cancer Gene Ther. 2020 Sep 12;28(5):442–54. doi: 10.1038/s41417-020-00226-z (PMC8119244; doi:10.1038/s41417-020-00226-z)
Supplement: Supplementary file 1 — Supplementary figure Legends [file 41417_2020_226_MOESM1_ESM.pdf]

Supplementary Figure Legends:

Supplementary Figure 1: **Association of adenovirus particles with lymphocytes and erythrocytes**. TILT-123 was incubated with human lymphocytes and erythrocytes at different VP/cell ratios at 37°C. After 30 min incubation, cellular fractions were isolated through centrifugation and analyzed through qPCR. Viral copy number was normalized against amount of genomic DNA in the sample, determined by the expression level of human β-actin. Data are presented as mean + SEM.

Supplementary Figure 2**: Adenovirus transduction after interaction with erythrocytes and lymphocytes.** Ad5/3-Luc1 was incubated at 37°C for 30 minutes with human erythrocytes (A) at 0.036 VP/cell or with human lymphocytes (B) at 10 VP/cell. Erythrocytes or lymphocytes alone in A549 cells and erythrocytes or lymphocytes with Ad5/3-Luc1 in the absence of A549 cells were used as negative controls. Ad5/3-Luc1 alone with A549 cells used as positive control. Luciferase expression was measured after 48 h. Data are presented as mean + SEM.

Supplementary Figure 3 **-Adenovirus transduction and tumor cell killing potential after interaction with lymphocytes and erythrocytes.** A549 cells were plated onto the bottom well of a Transwell plate 24 hours before the experiment. TILT-123 was incubated at 37°C for 30 minutes with human lymphocytes at 10 VP/cell or with human erythrocytes at 0.036 VP/cell. After incubation, samples were centrifuged, resuspended and then 300 ul of cell-adenovirus mixture was added on the Transwell for 4 hours. Tumor-killing ability of TILT-123 as such (0.1-100 VP/A549 cell) or when delivered with lymphocytes or erythrocytes was analyzed on day 3 with cytotoxicity (MTS) assay on A549 cells. Data are presented as mean + SEM.

Supplementary Figure 4 **-Adenovirus biodistribution in spleens and serum in the presence or absence of lymphocytes and erythrocytes.** Immunodeficient NMRI mice bearing subcutaneous A549 human lung adenocarcinoma tumors were injected intravenously with 2 X 10e9 VP/100 µl of TILT-123 alone or previously incubated with or without either human lymphocytes or erythrocytes (667 VP/cell) and with or without antiserum. Positive control and negative mock control received 2 X 10e10 VP/100 µl of TILT-123 and PBS, respectively. After 3 days, mice were euthanized. Spleens (A and C with cell-virus mixed with antiserum) and serum (B and D with cell-virus mixed with antiserum) samples were collected to detect adenovirus Ad5/3 genome through qPCR. Viral copy number was normalized against amount of genomic DNA in the sample, determined by the expression level of mouse β-actin. Data are presented as mean.

Supplementary figure 5 **- Transgene expression after adenovirus systemic transduction of tumor in the presence or absence of lymphocytes and erythrocytes**. Immunodeficient NMRI mice bearing subcutaneous A549 lung adenocarcinoma tumors were injected intravenously with 2 X 10e9 VP/100 µl of TILT-123 alone or previously incubated with or without human lymphocytes or erythrocytes (667 VP/cell) and with or without anti-serum. Positive control and negative mock control received 2 X 10e10 VP/100 µl of TILT-123 and PBS, respectively. After 3 days, mice were euthanized and tumors were collected to detect expression of hIL-2 (A, C) and hTNF-α (B, D). Cytokine concentrations were normalized against the total protein contents of the sample.
