# Supplementary figures and images for: Ad5/3 is able to avoid neutralization by binding to erythrocytes and lymphocytes
Source: Cancer Gene Ther. 2020 Sep 12;28(5):442–54. doi: 10.1038/s41417-020-00226-z (PMC8119244; doi:10.1038/s41417-020-00226-z)

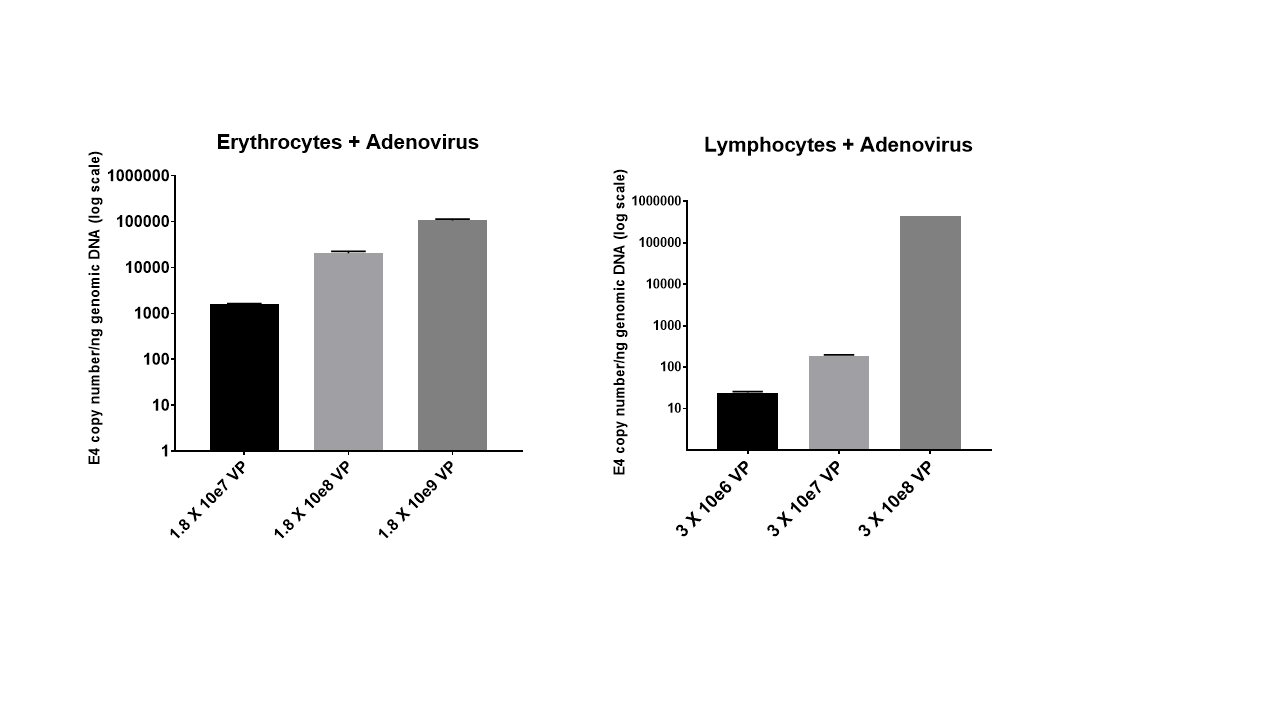

Supplement: Supplementary file 2 — Supplementary figure 1 [file 41417_2020_226_MOESM2_ESM.tif]

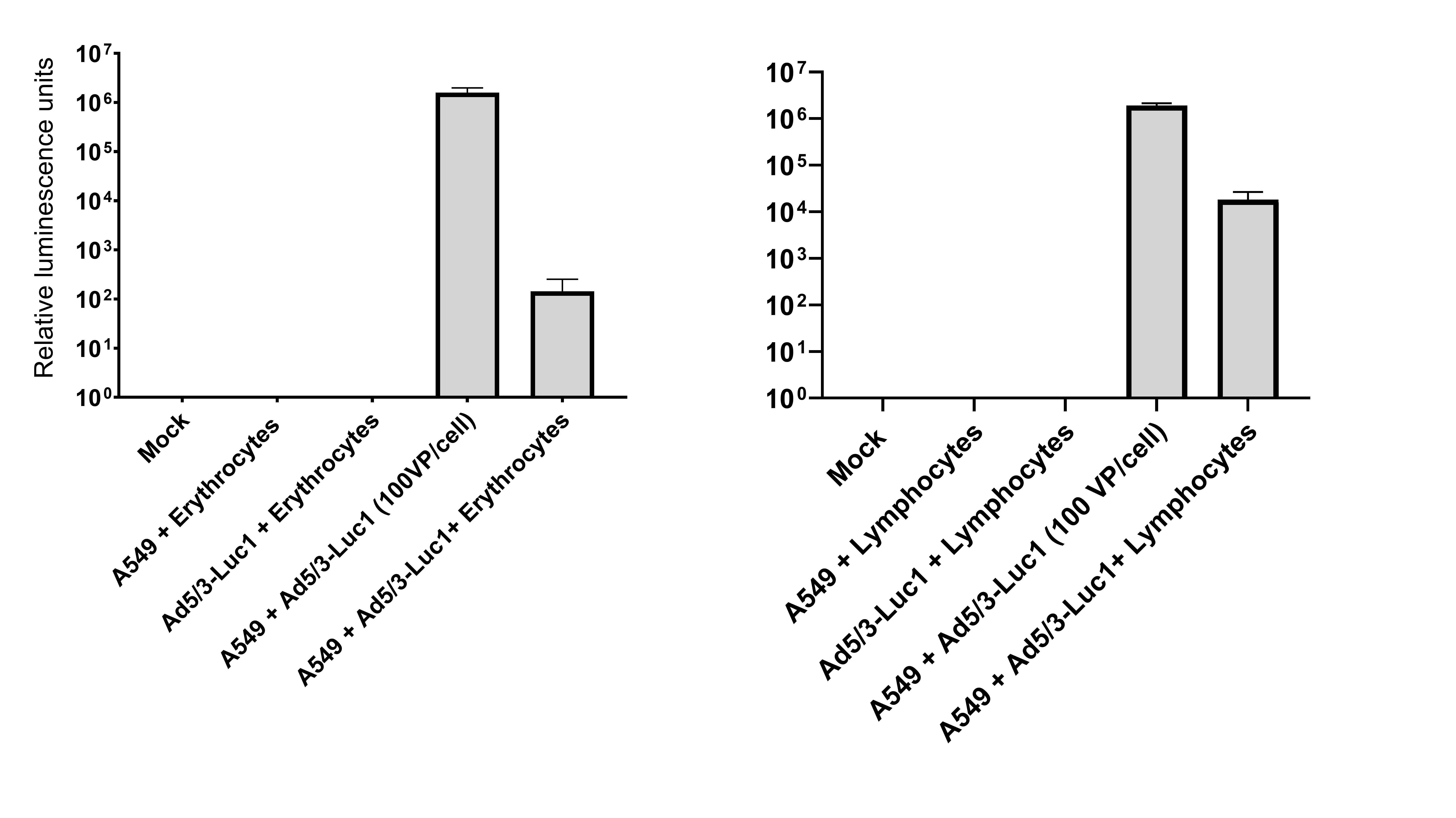

Supplement: Supplementary file 3 — Supplementary figure 2 [file 41417_2020_226_MOESM3_ESM.tif]

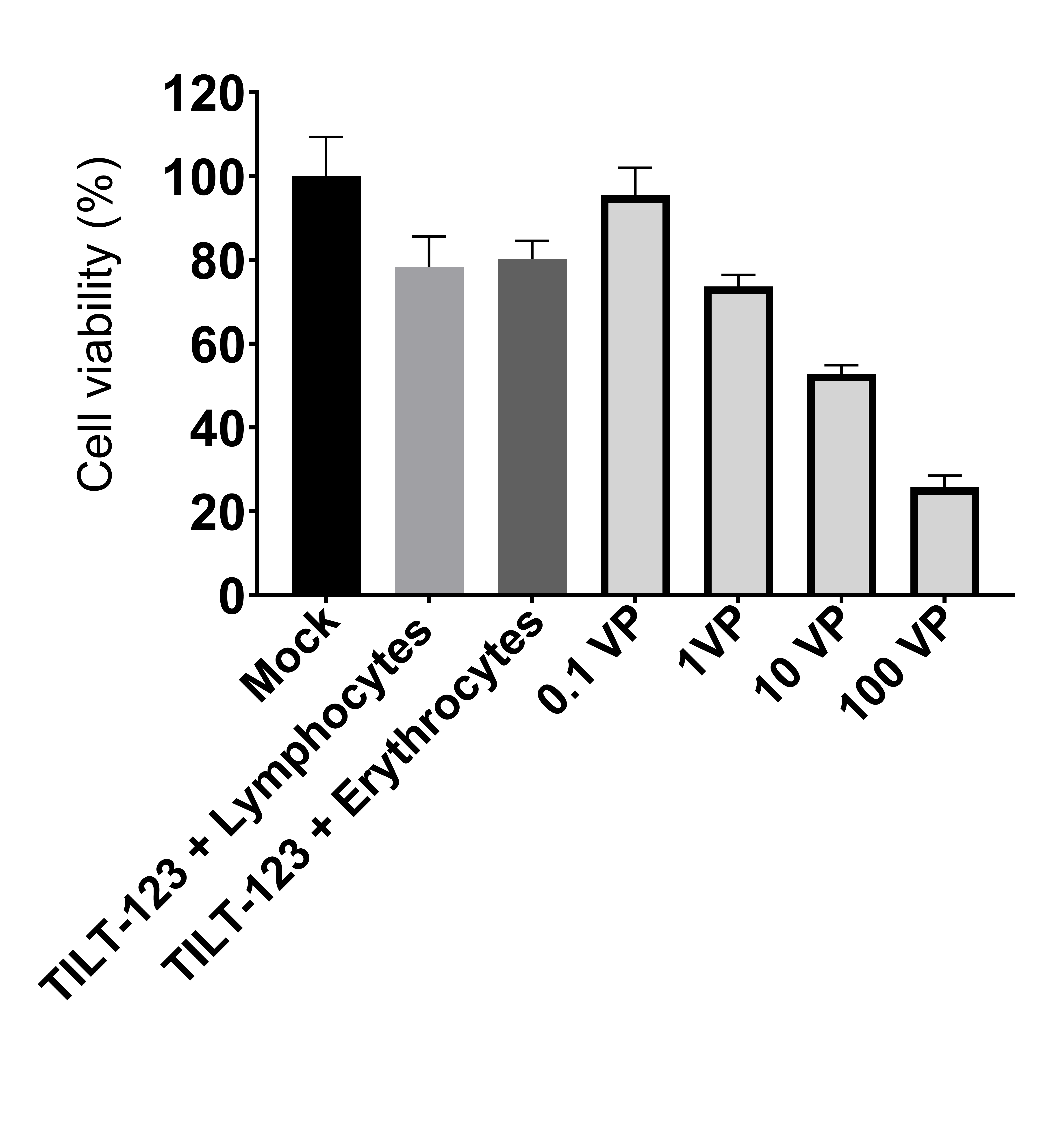

Supplement: Supplementary file 4 — Supplementary figure 3 [file 41417_2020_226_MOESM4_ESM.tif]

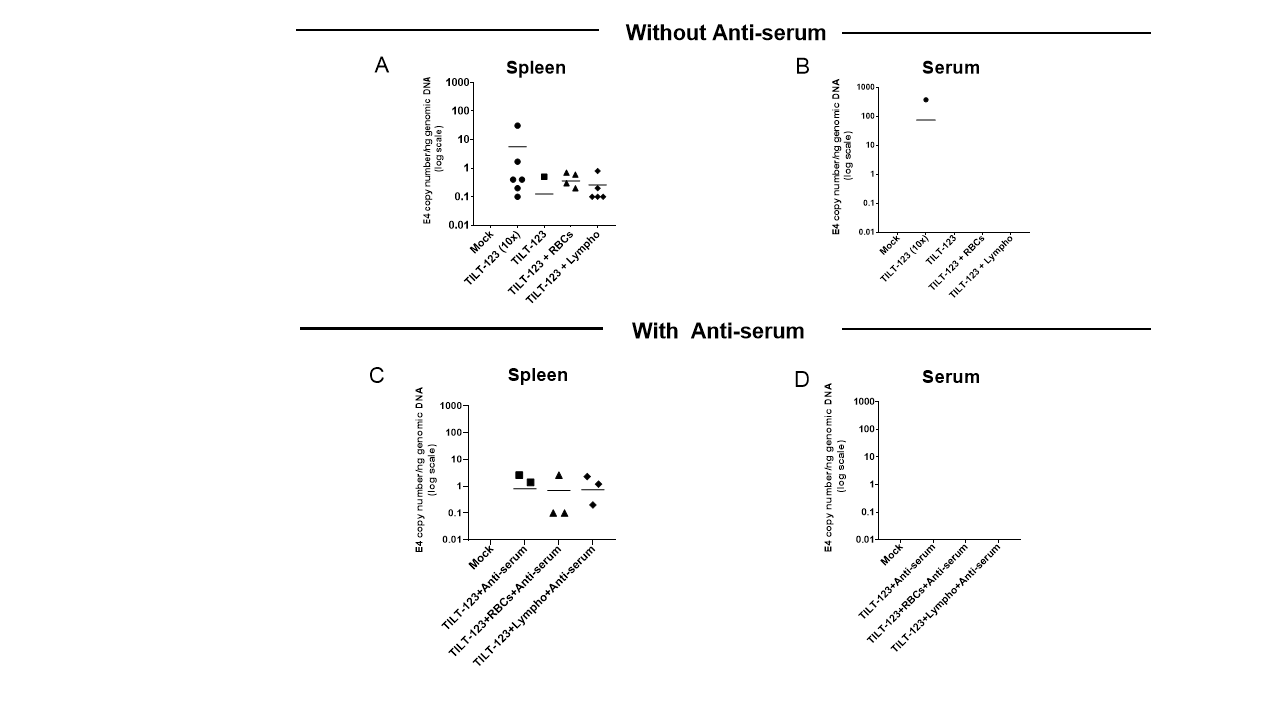

Supplement: Supplementary file 5 — Supplementary figure 4 [file 41417_2020_226_MOESM5_ESM.tif]

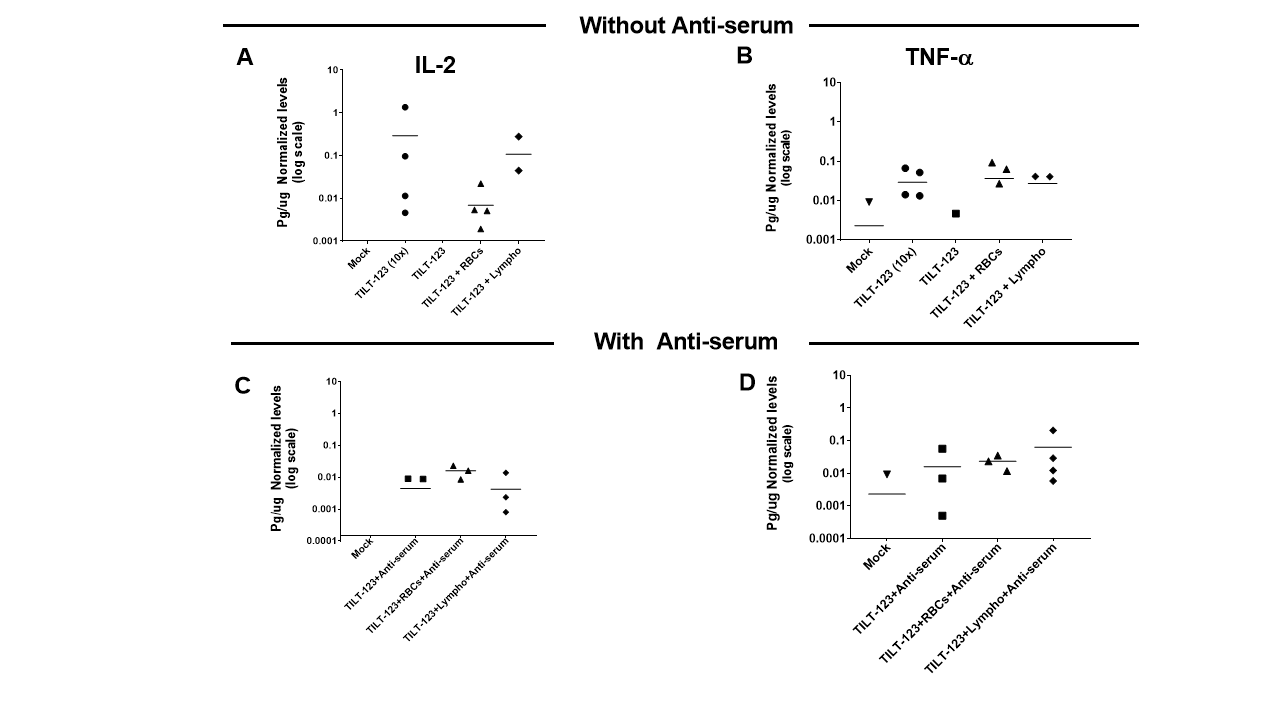

Supplement: Supplementary file 6 — Supplementary figure 5 [file 41417_2020_226_MOESM6_ESM.tif]
